# The effect of parathyroid hormone lowering by etelcalcetide therapy on calcification propensity and calciprotein particles in hemodialysis patients

**DOI:** 10.1093/ckj/sfae097

**Published:** 2024-03-30

**Authors:** Ursula Thiem, Jakob Lenz, Maria C Haller, Andreas Pasch, Edward R Smith, Daniel Cejka

**Affiliations:** Department of Medicine III - Nephrology, Hypertension, Transplantation Medicine, Rheumatology, Geriatrics, Ordensklinikum Linz - Elisabethinen Hospital, Linz, Austria; Department of Medicine III - Nephrology, Hypertension, Transplantation Medicine, Rheumatology, Geriatrics, Ordensklinikum Linz - Elisabethinen Hospital, Linz, Austria; Department of Medicine III - Nephrology, Hypertension, Transplantation Medicine, Rheumatology, Geriatrics, Ordensklinikum Linz - Elisabethinen Hospital, Linz, Austria; CeMSIIS - Center for Medical Statistics, Informatics, and Intelligent Systems, Medical University Vienna, Vienna, Austria; Calciscon AG, Biel, Switzerland; Lindenhofspital Bern, Bern, Switzerland; Department of Physiology and Pathophysiology, Johannes Kepler University Linz, Linz, Austria; Department of Nephrology, The Royal Melbourne Hospital, Parkville, Victoria, Australia; Department of Medicine (RMH), The University of Melbourne, Parkville, Victoria, Australia; Department of Medicine III - Nephrology, Hypertension, Transplantation Medicine, Rheumatology, Geriatrics, Ordensklinikum Linz - Elisabethinen Hospital, Linz, Austria

**Keywords:** calcification propensity, calcimimetics, calciprotein particles, chronic hemodialysis, secondary hyperparathyroidism

## Abstract

**Background:**

This study investigated whether parathyroid hormone (PTH) lowering with etelcalcetide, and the consequent effects on mineral and bone metabolism, could improve serum calcification propensity (T50 time) and decrease calciprotein particle (CPP) load in hemodialysis patients with secondary hyperparathyroidism.

**Methods:**

In this single-arm, prospective, dose-escalation proof-of-principle study, hemodialysis patients received etelcalcetide at 2.5 mg/dialysis session with increments of 2.5 mg every 4 weeks to a maximum dose of 15 mg three times a week or until a pre-specified safety endpoint was reached, followed by an 8-week wash-out phase.

**Results:**

Out of 36 patients recruited (81% male, 62 ± 13 years), 16 patients completed the study per protocol with a mean maximum tolerated dose of etelcalcetide of 9.5 ± 2.9 mg/dialysis session. With escalating doses of etelcalcetide, PTH and serum calcium levels significantly decreased (*P *< 0.0001). While there was no significant change in T50 times or serum phosphate levels, etelcalcetide did yield significant and consistent reductions in serum levels of endogenous calciprotein monomers [−35.4 (−44.4 to −26.5)%, *P *< 0.0001], primary [−22.4 (−34.5 to −10.3)%, *P *< 0.01] and secondary CPP [−29.1 (−45.7 to −12.4)%, *P *< 0.01], an effect that was reversed after therapy withdrawal. Serum levels of osteoclastic markers significantly decreased with escalating doses of etelcalcetide, while levels of the osteoblastic marker remained stable.

**Conclusions:**

Lowering of PTH with etelcalcetide did not result in statistically significant changes in T50. By contrast, homogenous reductions in serum levels of calciprotein monomers, primary and secondary CPP were observed.

KEY LEARNING POINTS **What was known**:

- Etelcalcetide is a potent second-generation calcimimetic used in dialysis patients with secondary hyperparathyroidism, which is highly prevalent in these patients and associated with the development of abnormal bone turnover, vascular calcification, and an increased cardiovascular morbidity and mortality.
- The efficacy of parathyroid hormone lowering with the calcimimetic etelcalcetide, and the consequent effects on mineral and bone metabolism, at improving serum calcification propensity (T50 time) and lowering endogenous calciprotein particle levels in dialysis patients is insufficiently studied.

**This study adds**:

- In this single-arm, prospective pilot study in dialysis patients, parathyroid hormone and serum calcium levels significantly decreased under escalating doses of etelcalcetide, an effect that was reversed after drug withdrawal.
- Etelcalcetide had no effect on blood calcification propensity, only a non-significant trend for increases in T50 times with escalating doses of etelcalcetide was observed.
- Etelcalcetide therapy consistently reduced serum levels of endogenous calciprotein monomers, primary and secondary calciprotein particles in stable hemodialysis patients with secondary hyperparathyroidism.

**Potential impact**:

- If T50 times or calciprotein particles could provide additional information to guide therapy in patients with secondary hyperparathyroidism requires further investigation.

## INTRODUCTION

Secondary hyperparathyroidism (sHPT) is highly prevalent in patients with end-stage kidney disease and is associated with the development of abnormal bone turnover, vascular calcification, and an increased cardiovascular morbidity and mortality[1–4]. Etelcalcetide is a potent second-generation calcimimetic used in the treatment of sHPT in hemodialysis patients [5–7], which lowers parathyroid hormone (PTH) levels and achieves sustained reductions in serum calcium (sCa) and phosphate (sPh). Etelcalcetide therapy is also associated with decreased levels of osteoblastic and osteoclastic bone turnover markers in these patients[5–7]. Phosphate, and to a lesser extent calcium, are key determinants of blood calcification propensity, which can be assessed *in vitro* by the T50 test[8]. The T50 test measures crystal formation time, i.e. transition time from primary amorphous calciprotein particles (CPP-I) to secondary crystalline calciprotein particles (CPP-II) after supersaturation of patients’ sera with calcium and phosphate *ex vivo*. Lower T50 levels reflect a higher blood calcification propensity and were independently associated with the composite end point all-cause mortality, myocardial infarction, and peripheral vascular events in a *post hoc* analysis of the EVOLVE study, which investigated the effect of PTH lowering with the first-generation calcimimetic cinacalcet in dialysis patients with sHPT[9]. Longitudinal T50 testing under calcimimetic therapy, however, was not performed in this trial. In contrast to T50, which is an *in vitro* test of mineral buffering capacity of serum, calciprotein particles (CPPs) are also formed endogenously and can be detected in blood of healthy people and patients with chronic kidney disease[10–12] and may reflect the spent mineral buffering capacity in blood. CPPs have been found to induce endothelial damage and inflammation *in vivo* and potentiate calcification of vascular smooth muscle cells *in vitro*[11, 13–17].

We hypothesized that in chronic hemodialysis patients with sHPT, lowering PTH with etelcalcetide and the resultant lowering of sCa, sPh, and suppression of bone turnover, would improve the resistance of blood toward calcification, as reflected by an increase in T50. Furthermore, the effect of etelcalcetide on endogenous calciprotein monomer (CPM), CPP-I, and CPP-II serum levels was explored.

## MATERIALS AND METHODS

### Design

This single-arm, prospective, dose-escalation pilot study in stable hemodialysis patients with sHPT was conducted at the dialysis facility of the Ordensklinikum Linz Elisabethinen Hospital between May 2019 and March 2022. It was approved by the Ethics Committee of Upper Austria (1138/2018) and conducted according to regulations of the International Conference on Harmonization on Good Clinical Practice and the Declaration of Helsinki. All study participants gave written informed consent. This study was registered before initiation at the European Union Clinical Trials Register (EUDRACT 2018–000421-31) and ClinicalTrials.gov (NCT03795558).

Initially, there was a 4‒12-week run-in/wash-out phase without the use of calcimimetics (cinacalcet or etelcalcetide) similar to previous studies[5, 7] to allow for increases in PTH >9× upper limit of normal (ULN). Study visits were held every 4 weeks to monitor the levels of PTH and sCa_albumin-corrected_. If PTH levels did not reach >9× ULN and simultaneously sCa_albumin-corrected_ levels were not ≥2.08 mmol/l within 12 weeks of the run-in/wash-out phase, patients were excluded from further participation in the study and medical treatment was continued according to local practice. Once patients presented with PTH levels >9× ULN, they received etelcalcetide intravenously at a starting dose of 2.5 mg/dialysis session. According to the label, etelcalcetide dose was escalated every 4 weeks in 2.5 mg/dialysis session increments to a maximum dose of 15 mg three times a week in a fixed sequence dose-escalation protocol until the end of study or until a pre-specified safety endpoint was reached following the approach used by Block *et al.*[5, 7] (sCa_albumin-corrected _< 1.88 mmol/l, PTH < 100 pg/ml or symptomatic hypocalcemia). After completion of the 15 mg three times a week phase or if a pre-specified safety endpoint was reached, etelcalcetide was discontinued and patients were followed for another 8 weeks to study any potential reversibility of PTH lowering on T50.

Study visits took place at the beginning of each study phase and 1 week thereafter. Blood was drawn at every study visit at the first dialysis session of the week immediately before initiation of hemodialysis via the dialysis access.

### Endpoint

Etelcalcetide dose-response curves of PTH and T50 and their association were tested. We hypothesized that this association could be either curvilinear (the lower the PTH levels, the longer the T50 times) or bell-shaped (both very low and very high PTH levels are paralleled by lower T50 times). Further exploratory analysis included the effect of etelcalcetide on CPPs, fibroblast growth factor-23 (FGF-23), and bone turnover marker levels.

### Participants

Eligibility criteria are listed in Table 1.

**Table 1:** Eligibility criteria.

Inclusion criteria
Age ≥18 years
Chronic (≥3 months) treatment with hemodialysis or hemodiafiltration three times a week
Secondary hyperparathyroidism (PTH ˃9× ULN if therapy-naïve or PTH >2× ULN with current oral calcimimetics use)
Albumin corrected serum calcium ≥2.08 mmol/l
Stable calcium concentration of the dialysis bath for at least 2 weeks prior to screening
**Exclusion criteria**
Ongoing etelcalcetide therapy
Known allergy to etelcalcetide
History of parathyroidectomy or parathyroidectomy expected
Therapy with bisphosphonates within the past 12 months or with denosumab within the past 6 months
Current use of antacids containing aluminum, calcium, magnesium, or bicarbonate
History of myocardial infarction, coronary angioplasty or coronary arterial bypass grafting within the past 6 months
History of symptomatic ventricular arrhythmias or Torsades des Pointes
Pregnancy

### Safety

Levels of sCa were measured at each study visit (before dose escalation and 1 week thereafter). In case of sCa_albumin-corrected _< 1.88 mmol/l or PTH < 100 pg/ml or symptomatic hypocalcemia, etelcalcetide was discontinued and patients were followed for an additional 8 weeks. In case of hypocalcemia or hypocalcemia-associated symptoms, an electrocardiogram was to be performed to study the QTc time. Rescue therapy (10 ml of 10% calcium gluconate intravenously over 10 minutes) and subsequent maintenance of calcium levels with oral calcium or vitamin D supplements, calcium-containing phosphate binders or increased dialysate calcium was protocolized for patients with QTc times >500 ms, but not observed in any patient.

In case of gastrointestinal symptoms (nausea, vomiting, diarrhea) not tolerated by the patient, etelcalcetide was also discontinued.

### Concomitant medication and dialysis prescription

Concomitant medication that might potentially influence calcification propensity (oral phosphate binders, active vitamin D), dialysis mode and schedule (hemodialysis or hemodiafiltration, prescribed dialysis duration, blood and dialysate flow), and concentrations of magnesium, calcium, and bicarbonate in the dialysate were held constant during the study as far as clinically justifiable. As decreases in sCa with etelcalcetide treatment were expected and countermeasures (oral calcium supplementation, increases in calcium concentration of dialysate, treatment with active vitamin D) might influence calcification propensity, no attempt was made to correct decreasing calcium levels unless patients would have developed long QT or symptomatic hypocalcemia.

### Biochemical analyses

Routine blood tests were performed in the central laboratory facility of the Ordensklinikum Linz Elisabethinen Hospital using Cobas analyzer systems (Roche Diagnostics, Rotkreuz, Switzerland) without knowledge of the study phase. Samples which were not immediately analyzed in the central laboratory were centrifuged at 1280*g* for 15 minutes at room temperature with aliquots stored at −80°C until the time of testing. Technical details of the assays used are summarized in Supplementary Table 1.

### Statistical analysis

As pre-defined in the study protocol, all analyses were performed using the per protocol population, i.e. patients treated with etelcalcetide for at least 4 weeks who had two blood drawings for the determination of T50 after this treatment period. As this was a proof-of-concept pilot study and as no data from previous studies had been available at the time of the conception of the study to inform the process of sample size calculation, a formal sample size calculation was not performed and all statistical analyses are descriptive and exploratory. Laboratory parameters were summarized using descriptive statistics for each etelcalcetide dose. For three-group comparison, i.e. baseline versus maximum tolerated dose (MTD) of etelcalcetide versus wash-out, repeated measure ANOVA was used or, in case of missing values, a mixed model was fitted, and Tukey's test was used to adjust for multiple comparisons. To explore the relationship between changes in T50 and PTH, sPh and sCa, the slopes of the etelcalcetide dose-response curves including all measurements from baseline (after wash-out) to the MTD were derived by linear regression for each parameter. The relationship between slopes were then evaluated using Spearman's correlation. For statistical analyses and graphical illustration GraphPad Prism v.8.4.2 (GraphPad Software, Boston, MA, USA) was used.

## RESULTS

### Study population

**Figure 1:**
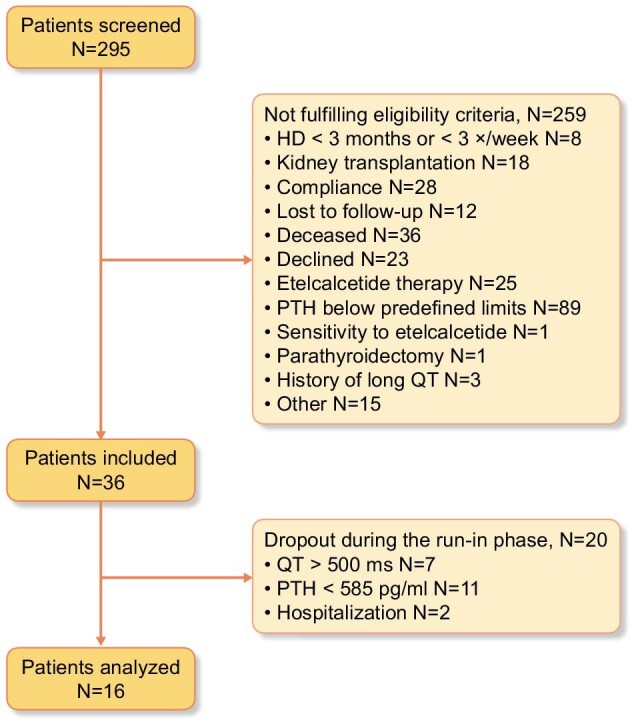
Patient flow chart. HD, hemodialysis.

Of 295 screened patients, 36 were included and 16 completed the study per protocol (Fig. 1). Screening failures and drop-out rates were unexpectedly high and the conduct of the study was severely compromised by the COVID-19 pandemic. Therefore, the study was closed after inclusion of 36 patients for feasibility reasons. Participants were predominantly male and exclusively of Caucasian ethnicity. Markers of chronic kidney disease-mineral and bone disorder were well controlled (Table 2).

**Table 2:** Patient characteristics at inclusion.

Characteristics	Overall population (*N* = 36)	Patients analyzed (*N* = 16)
Age (years)	62 ± 13	60 ± 16
Sex (male), *n* (%)	29 (81)	12 (75)
Dialysis vintage (months)	21 (11–43)	23 (13–58)
Primary kidney disease, *n* (%)		
Diabetes mellitus	7 (19.4)	2 (12.5)
Hypertensive/vascular	7 (19.4)	1 (6.3)
Glomerulonephritis	5 (13.9)	4 (25)
Polycystic kidney disease	3 (8.3)	1 (6.3)
Other	14 (38.9)	8 (50)
Biochemical parameters		
Serum bicarbonate (mmol/l)	20.95 ± 2.37	21.17 ± 2.69
Serum phosphate (mmol/l)	1.92 ± 0.47	1.99 ± 0.49
Albumin corrected serum calcium (mmol/l)	2.12 ± 0.14	2.14 ± 0.14
Serum albumin (g/dl)	3.89 ± 0.31	3.93 ± 0.30
Intact parathyroid hormone (pg/ml)	302 (242–466)	465 (262–622)
C-reactive protein (mg/dl)	0.46 (0.23–1.14)	0.37 (0.16–1.05)
Comorbidities, *n* (%)		
Arterial hypertension	34 (94.4)	16 (100)
Diabetes mellitus	13 (36.1)	4 (25)
Coronary artery disease	19 (52.8)	9 (56)
History of myocardial infarction	5 (13.9)	2 (12.5)
Chronic heart failure	24 (66.7)	10 (63)
Atrial fibrillation	5 (13.9)	2 (12.5)
Peripheral arterial disease	11 (30.6)	4 (25)
History of amputation	3 (8.3)	2 (12.5)
Cerebral arterial disease	6 (16.7)	2 (12.5)
History of stroke	5 (13.9)	1 (6.3)
Smoker	5 (13.9)	2 (12.5)
Medication, *n* (%)		
Sevelamer carbonate	31 (86.1)	13 (81.3)
Lanthanum carbonate	4 (11.1)	2 (12.5)
Sucroferric oxyhydroxide	2 (5.6)	1 (6.3)
Vitamin K antagonist	3 (8.3)	2 (12.5)
Active vitamin D	29 (80.6)	14 (87.5)
Native vitamin D	4 (11.1)	1 (6.3)
Cinacalcet	34 (94.4)	15 (93.8)

Data are presented as mean ± standard deviation or median (interquartile range), according to distribution.

### Effect of etelcalcetide on blood calcification propensity (T50), parathyroid hormone, serum calcium, and phosphate levels

**Figure 2:**
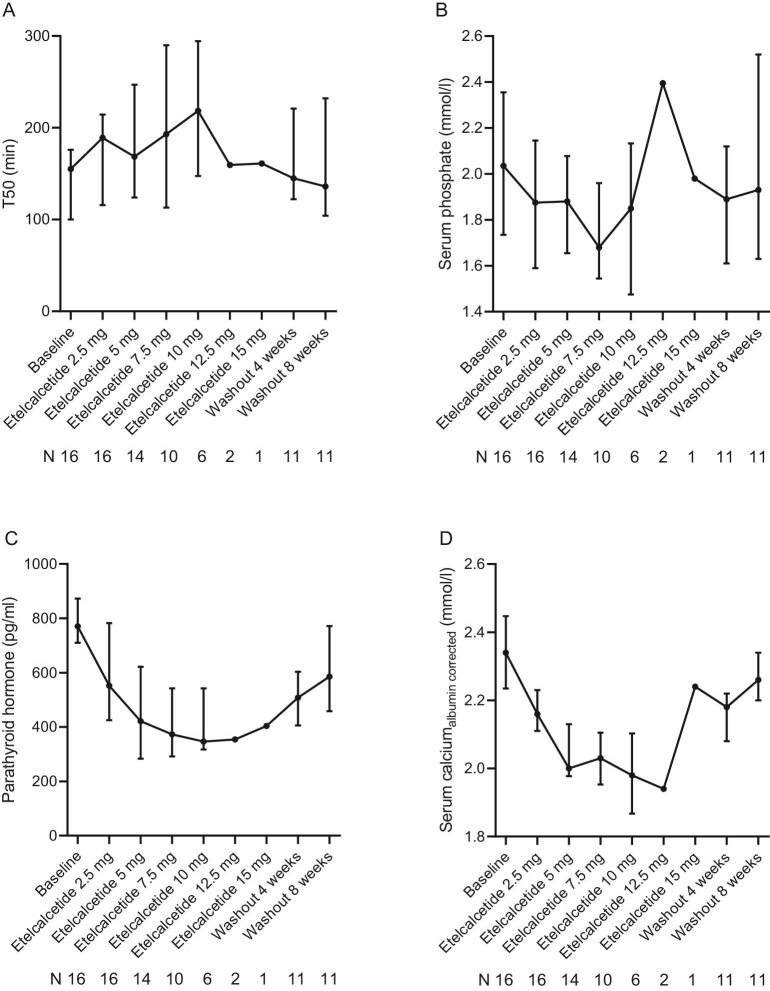
Time course of T50, PTH, serum calcium, and phosphate levels with escalating doses of etelcalcetide and wash-out. Chronic hemodialysis patients with secondary hyperparathyroidism were treated with escalating doses of etelcalcetide starting after a 4‒12-week run-in/wash-out phase without the use of calcimimetics (baseline) at a dose of 2.5 mg intravenously per dialysis session. Etelcalcetide dose was escalated every 4 weeks in 2.5 mg/dialysis session increments to a maximum dose of 15 mg three times a week until the end of study or until a pre-specified safety endpoint was reached. After completion of the 15 mg three-times-weekly phase or in case a safety endpoint was reached, etelcalcetide was discontinued and patients were followed for another 8 weeks (wash-out weeks 4 and 8). Data are presented as medians and interquartile ranges.

**Figure 3:**
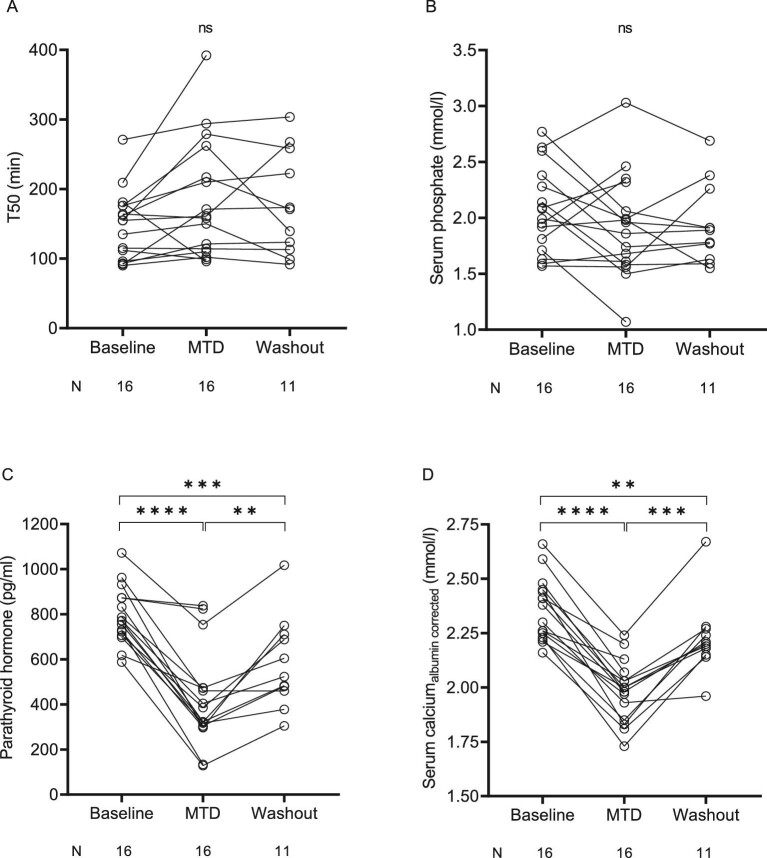
Effect of etelcalcetide on T50, PTH, serum calcium, and phosphate levels. Chronic hemodialysis patients with secondary hyperparathyroidism were treated with escalating doses of etelcalcetide starting at a dose of 2.5 mg per dialysis session with increments of 2.5 mg per dialysis session every 4 weeks to a maximum dose of 15 mg three times a week or until a pre-specified safety endpoint was reached. After completion of the 15 mg three-times-weekly phase or in case a safety endpoint was reached, etelcalcetide was discontinued and patients entered an 8-week wash-out phase. Data were analyzed by fitting a mixed model and Tukey's multiple comparison test was used for *post hoc* comparisons between baseline values (i.e. after a 4‒12-week run-in/wash-out phase without the use of calcimimetics), the MTD of etelcalcetide, and etelcalcetide wash-out (i.e. mean of all available values within 8 weeks after etelcalcetide withdrawal). ns, not significant, **P < 0.01, ***P < 0.001, ****P < 0.0001.

The mean MTD of etelcalcetide was 9.5 ± 2.9 mg. T50 times tended to increase and sPh levels tended to decrease with escalating doses of etelcalcetide (Fig. 2a, b), but changes did not reach statistical significance (*P *= 0.09 for T50, *P *= 0.30 for sPh, Fig. 3a, b). We observed a heterogenous response to etelcalcetide with respect to changes in T50 in patients despite comparable magnitudes of PTH lowering (Supplementary Fig. 1). Dose-response slopes for T50 inversely correlated with those of sPh (*r* = −0.797, *P *< 0.001), but not with PTH (*r* = -0.20, *P *= 0.49) or sCa (*r* = −0.415, *P *= 0.14) (Supplementary Fig. 2).

As expected, PTH and sCa levels decreased in parallel with escalating doses of etelcalcetide (Fig. 2c, d), an effect that reversed after drug withdrawal (Fig. 3c, d).

### Effect of etelcalcetide on calciprotein monomers and calciprotein particles

**Figure 4:**
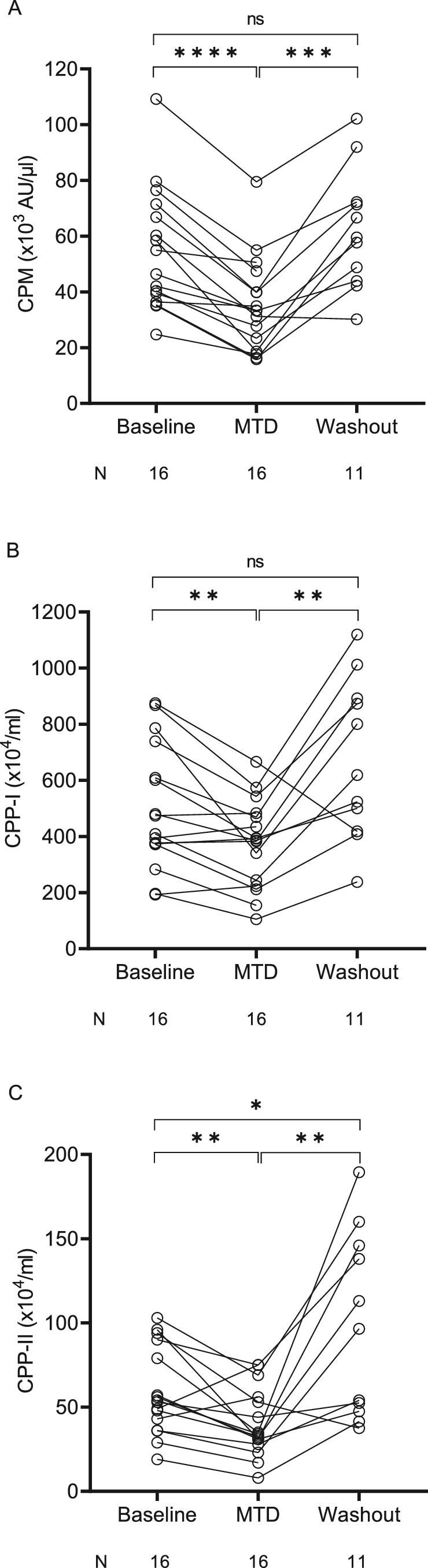
Effect of etelcalcetide on calciprotein monomers, primary and secondary CPPs. Chronic hemodialysis patients with secondary hyperparathyroidism were treated with escalating doses of etelcalcetide starting at a dose of 2.5 mg per dialysis session with increments of 2.5 mg per dialysis session every 4 weeks to a maximum dose of 15 mg three times a week or until a pre-specified safety endpoint was reached. After completion of the 15 mg three-times-weekly phase or in case a safety endpoint was reached, etelcalcetide was discontinued and patients entered an eight-week wash-out phase. Serum levels of (**a**) calciprotein monomers (CPM), (**b**) primary calciprotein particles (CPP-I), and (**c**) secondary calciprotein particles (CPP-II) were compared between baseline values (i.e. after a 4‒12-week run-in/wash-out phase without the use of calcimimetics), the MTD of etelcalcetide, and etelcalcetide wash-out (i.e. mean of all available values within 8 weeks after etelcalcetide withdrawal) by fitting a mixed model and Tukey's multiple comparison test was used for *post hoc* comparisons. ns, not significant, *P < 0.05, **P < 0.01, ***P < 0.001, ****P < 0.0001.

Etelcalcetide therapy significantly reduced the load of CPM [mean −35.4 (95% CI −44.4 to −26.5)%], CPP-I [−22.4 (−34.5 to −10.3)%], and CPP-II [−29.1 (−45.7 to −12.4)%], an effect that reversed after withdrawal of etelcalcetide (Fig. 4a‒c, Table 3). We found significant associations between the dose-response slopes for sPh and CPP-II (*r* = 0.6093, *P *< 0.05), sCa and CPM (*r* = 0.5912, *P *< 0.05), CPP-II (*r* = 0.6167, *P *< 0.05, Supplementary Fig. 3), and PTH and CPP-I (*r* = 0.7582, *P *< 0.01).

**Table 3:** Effect of etelcalcetide on calciprotein monomers, primary, and secondary CPPs.

Parameter	Baseline	MTD of etelcalcetide	Wash-out
CPM (AU/µl)	50.7 (37.2–70.3) × 10^3^	33.3 (19.8–45.6) × 10^3^	59.6 (43.9–72.3) × 10^3^
CPP-I (particles/ml)	441.5 (372.5–706.5) × 10^4^	388.5 (228.3–479.5) × 10^4^	619.0 (418.5–892.5) × 10^4^
CPP-II (particles/ml)	53.5 (37.8–87.3) × 10^4^	33.5 (28.8–55.3) × 10^4^	96.5 (47.5–146.0) × 10^4^

CPM, calciprotein monomers; CPP-I, primary calciprotein particles; CPP-II, secondary calciprotein particles

### Effect of etelcalcetide on bone turnover markers and intact fibroblast growth factor-23

**Figure 5:**
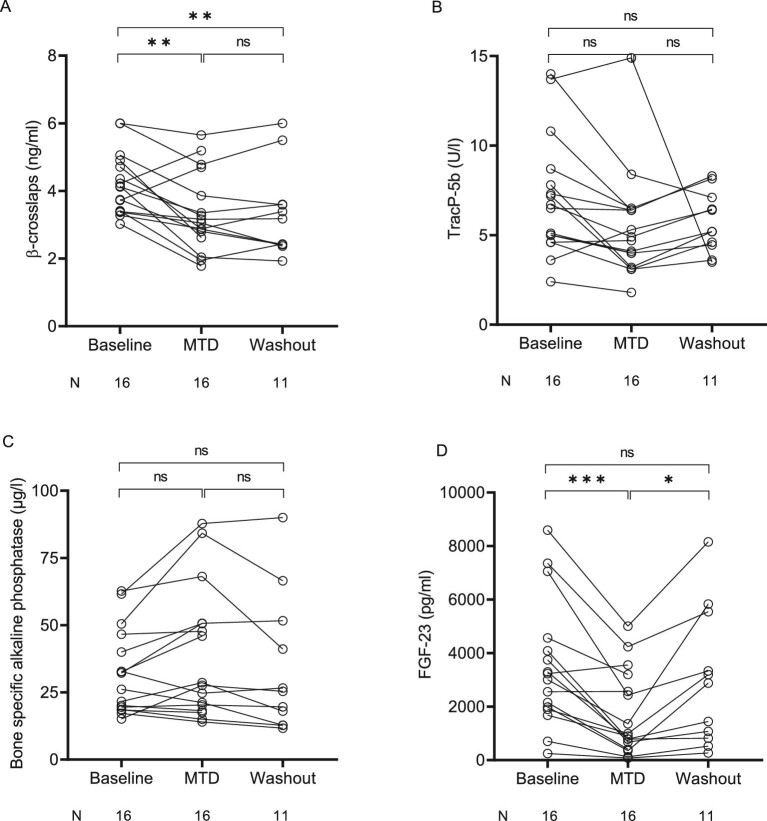
Effect of etelcalcetide on bone turnover markers and fibroblast growth factor-23. Chronic hemodialysis patients with secondary hyperparathyroidism were treated with escalating doses of etelcalcetide starting at a dose of 2.5 mg per dialysis session with increments of 2.5 mg per dialysis session every 4 weeks to a maximum dose of 15 mg three times a week or until a pre-specified safety endpoint was reached. After completion of the 15 mg three-times-weekly phase or in case a safety endpoint was reached, etelcalcetide was discontinued and patients entered an 8-week wash-out phase. Serum levels of (**a**) β-crosslaps, (**b**) tartrate-resistant acid phosphatase 5b (TRAcP-5b), (**c**) bone specific alkaline phosphatase, and (**d**) fibroblast growth factor-23 (FGF-23) were compared between baseline values (i.e. after a 4‒12-week run-in/wash-out phase without the use of calcimimetics), the MTD of etelcalcetide, and etelcalcetide wash-out (i.e. mean of all available values within 8 weeks after etelcalcetide withdrawal) by fitting a mixed model and Tukey's multiple comparison test was used for *post hoc* comparisons. ns, not significant, *P < 0.05, **P < 0.01, ***P < 0.001.

The effects of etelcalcetide on bone turnover markers are shown in Fig. 5a‒d. Serum levels of β-crosslaps (CTX), an osteoclastic marker, significantly decreased with escalating doses of etelcalcetide (from 4.22 ± 0.92 ng/ml at baseline to 3.37 ± 1.17 ng/ml under MTD of etelcalcetide, *P *< 0.01). Plasma levels of TracP-5b, another osteoclastic marker, tended to decrease but did not reach statistical significance [6.6 (4.7–8.5) U/l versus 4.8 (3.4–6.4) U/l at baseline and under the MTD of etelcalcetide, respectively, *P *= 0.1088]. Serum intact FGF-23 levels significantly decreased under etelcalcetide [3117 (1919–4444) pg/ml versus 944 (471–3042) pg/ml, *P *< 0.001] and returned to baseline after wash-out [2877 (817–5546) pg/ml]. Etelcalcetide had no effect on serum levels of the osteoblastic marker bone specific alkaline phosphatase.

### Safety

In 10 patients of the per protocol population (*N* = 16), etelcalcetide was stopped according to the protocol because of low sCa or PTH levels (*N* = 5 at 7.5 mg, *N* = 3 at 10 mg, and *N* = 2 at 12.5 mg of etelcalcetide). Only one patient tolerated etelcalcetide at a dose of 15 mg three times a week. No episodes of symptomatic hypocalcemia occurred during the study.

In the overall population (*N* = 36), 37 adverse events were reported in 24 patients, most of which was mild and did not require interventions (Table 4). There were nine serious adverse events, none of which was judged to be related to the study drug (Table 5). Approximately 70% of all these events occurred during the run-in and wash-out phases.

**Table 4:** Summary of reported adverse events.

Mild adverse events	Study phase	*N*
QTc prolongation	run-in phase	7
Ventricular bigeminy	run-in phase	1
Hypotension during dialysis	run-in phase *N* = 1	3
	treatment phase *N* = 2	
Chest pain	treatment phase	1
Dyspnea	wash-out phase	1
Abdominal pain	run-in phase	1
Diarrhea	run-in phase	1
Nausea	run-in phase	1
COVID-19	treatment phase	1
Weakness	wash-out phase	1
Vertigo	wash-out phase	1
Paresthesia	wash-out phase	1
Muscle or joint pain	run-in/wash-out phase *N* = 4	6
	treatment phase *N* = 2	
Bursitis	run-in phase	1
Skin hematoma	wash-out phase	1
Pruritus	wash-out phase	1
Skin alterations	run-in phase *N* = 1	2
	treatment phase *N* = 1	
Allergic conjunctivitis	treatment phase	1
Cataract	treatment phase	1
Moderate adverse events		*N*
Hypertensive crisis	run-in phase	1
Erysipelas	treatment phase	1
Progressive oliguria	run-in phase	1
Thrombosis of the arteriovenous fistula	treatment phase	1

**Table 5:** Serious adverse events.

Event	Study phase	*N*
Intraabdominal abscess	run-in phase	1
Worsening of pre-existing peripheral arterial vascular disease with the need for amputation	run-in phase	1
Comminuted fracture of the lower leg	run-in phase	1
Herpes zoster of C5/C6 dermatomes (arteriovenous fistula arm)	wash-out phase	1
Progression of coronary heart disease with the need for percutaneous transluminal coronary angioplasty	treatment phase	1
Kidney transplantation	treatment phase	1
Rupture of a kidney cyst	treatment phase	1
Fall-related thigh fracture	treatment phase	1
Vestibular vertigo	treatment phase	1

## DISCUSSION

In this pilot study we investigated the effect of PTH lowering by the calcimimetic etelcalcetide on blood calcification propensity and calciprotein particle levels in stable hemodialysis patients with sHPT. Lowering of PTH with etelcalcetide did not result in statistically significant changes in T50. Only a non-significant trend for increases in T50 with escalating doses of etelcalcetide was observed. In contrast, we observed homogenous reductions in serum levels of CPM, CPP-I, and CPP-II. As expected, PTH and sCa levels also significantly decreased under escalating doses of etelcalcetide.

So far, only two studies assessed the effect of etelcalcetide on calcification propensity by T50 in dialysis patients[18, 19]. Shoji *et al.* found that 12-month treatment with etelcalcetide improved T50 by 20 minutes compared with maxicalcitol, an active vitamin D analog, while achieving similar levels of PTH in both groups[19]. Thus, the treatment effect of etelcalcetide on T50 times was attributed to changes in sPh and sCa levels, which were lower throughout the study under etelcalcetide compared with maxicalcitol[19]. On the other hand, Dörr *et al.* did not observe any changes in T50 trajectories in chronic hemodialysis patients treated with either etelcalcetide or alfacalcidol, another active vitamin D analog[18]. In the latter study sPh levels did not differ between the etelcalcetide- and alfacalcidol-treated group, which might explain the null finding regarding T50 levels[18]. According to the authors, an increased use of phosphate binders in the alfacalcidol-treated group likely explains the similar courses of phosphate levels in the etelcalcetide and alfacalcidol group[18]. Also, in the study by Shoji *et al.* there were significant changes in concurrent therapy with almost one-third of patients starting to take alfacalcidol in the etelcalcetide group and >10% of patients starting to take oral calcimimetics in the alfacalcidol group, making it difficult to estimate the true effects of these drugs[19]. In the present study, comedication was kept as constant as possible. During etelcalcetide treatment phases, concurrent medication was changed in two patients only (in one patient the dose of sevelamer was increased, in another patient native vitamin D was stopped and sevelamer started).

In contrast to T50 time, we observed significant and consistent reductions in endogenous CPM and CPP serum levels in most patients under etelcalcetide. So far, there are only few data available on the effect of calcimimetic therapy on endogenous calciprotein particle levels. In a prospective observational study in 62 dialysis patients, CPP-I levels increased after cessation of cinacalcet and, similar to the present study, and changes in CPP levels were strongly associated with an increase in PTH and sCa levels[20]. By contrast, in a recent secondary analysis of an open-label, randomized study in 124 dialysis patients with mild sHPT, etelcalcetide therapy had no effect on serum CPP levels after 6 and 12 weeks compared to no use of etelcalcetide[21]. This might be explained by the fact that patients in our study had more severe sHPT and treatment effects of etelcalcetide were more pronounced. Moreover, as active vitamin D and calcium preparations were co-administered to correct hypocalcemia in the Japanese study, changes in sCa levels were less pronounced than in the present study [22], which might affect CPP levels as observed in previous studies[20, 23].

From previous studies it is known that phosphate is a much stronger determinant of T50 than calcium[8] and CPM/CPP are also more strongly correlated with phosphate than calcium[9, 10]. The finding that CPM/CPP correlate with sCa levels is surprising. We hypothesize that relatively large fluctuations in sCa levels under escalating doses of etelcalcetide account for the apparent association similar to what was observed in peritoneal dialysis patients[23].

In our study, none of the participants required calcium gluconate rescue therapy for symptomatic hypocalcemia or hypocalcemia-induced QTc prolongation. As suggested by current KDIGO guidelines, we chose not to treat mild and asymptomatic hypocalcemia to avoid inappropriate calcium loading[24]. Moreover, no negative signals were associated with persistently low sCa levels in cinacalcet-treated patients of the EVOLVE trial[25] and a recent association study in incident hemodialysis patients failed to detect an increased mortality in dialysis patients with low sCa levels[26]. In accordance with previous studies using calcimimetics [5, 7, 21, 27–30], etelcalcetide therapy was accompanied by significant reductions in serum levels of FGF-23 and also CTX. After cessation of etelcalcetide, FGF-23, CPM, CPP-I, and CPP-II returned to or in case of CPP-II even above baseline levels. Owing to the explorative nature of the present study, a causal association or directionality between levels of CPP, FGF-23, and bone turnover markers cannot be derived. From a biological point of view, however, it is plausible that etelcalcetide by lowering bone turnover leads to lower levels of sCa, sPh, and CPP[31]. Lower levels of CPP, which act as the phosphate stimulus to osteocytes/osteoblasts [32], may in turn lead to reduced production of FGF-23 in bone cells. Etelcalcetide may also directly act on bone cells expressing the calcium sensing receptor[33]. Notably, in this study blood samples were drawn non-fasting, thus serum levels of CPM and CPP should be regarded to originate both from the gastrointestinal tract[34] and bone[31]. Still, we observed consistent and distinct changes in serum levels of CPM and CPP, supporting the hypothesis that bone is a significant source of these particles in hemodialysis with bone disease. Given that high CPP levels have been associated with vascular stiffness[35] and calcification [36, 37], and an increased cardiovascular morbidity and mortality in patients with chronic kidney disease [12, 38], lowering CPP levels could contribute to improved outcomes of these patients.

Similar to what we observed in dialysis patients treated with the phosphate binder sucroferric oxyhydroxide [39], there is a considerable interindividual variability in responses of T50 times also to etelcalcetide: In some patients we did not observe changes in T50 times despite marked changes in PTH under etelcalcetide. Yet others showed substantial changes in T50 times even with relatively small changes in PTH. These findings could reflect pure change, but could also suggest that some patients might benefit more than others from treatment with phosphate binders or calcimimetics in terms of their calcification propensity. Both uncontrolled hyperparathyroidism and oversuppression of PTH are associated with adverse patient outcomes[3, 40–42]. This knowledge is reflected by the current chronic kidney disease-mineral and bone disorder treatment guidelines that recommend titrating PTH within a certain range (2–9× ULN), thereby avoiding excessively high or low PTH levels[24]. However, for the individual patient the optimal PTH target levels is difficult to determine as patients with end-stage renal disease show a high interindividual variability in PTH resistance through different mechanisms[43–45] and both high- and low-turnover bone disease can occur over a broad range of PTH levels[46]. Today, there is no parameter available that adequately describes the degree of PTH resistance in dialysis patients. If T50 times or CPP could provide additional information to guide therapy requires further investigation.

A specific feature of the study is the rigorous patient selection. Similar to the landmark studies on the effect of etelcalcetide on sHPT in hemodialysis patients [5, 7], our study had a calcimimetic-free run-in phase to identify patients with more severe sHPT as we assumed that only this subset of patients would tolerate the maximum titration of etelcalcetide dose. This approach ensures a reasonable level of confidence that data can be interpreted appropriately in this proof-of-principle study. However, this rigorous patient selection might also be viewed as a limitation of this study as data presented here may not be generalizable to the entire dialysis population, rather only applicable to patients with more severe sHPT. As a pilot study, all analyses presented here are exploratory, posing the main limitation to draw definitive conclusions.

Lowering of PTH with etelcalcetide did not result in statistically significant changes in T50. Only a non-significant trend for increases in T50 with escalating doses of etelcalcetide was observed. Our findings suggest that treatment with etelcalcetide lowers the load of endogenous CPM, CPP-I, and CPP-II in serum of hemodialysis patients.

## Supplementary Material

sfae097_Supplemental_File

## Data Availability

The data underlying this article will be shared upon reasonable request to the corresponding author.
