# Supplementary material for: The effect of parathyroid hormone lowering by etelcalcetide therapy on calcification propensity and calciprotein particles in hemodialysis patients
Source: Clin Kidney J. 2024 Mar 30;17(6):sfae097. doi: 10.1093/ckj/sfae097 (PMC11197474; doi:10.1093/ckj/sfae097)
Supplement: sfae097_Supplemental_File [file sfae097_supplemental_file.pdf]

SUPPLEMENTARY MATERIAL

Supplementary Table 1. Analytical procedures

**Parameter**	**Method**
Intact parathyroid hormone (serum)	Elecsys parathyroid hormone (1-84) assay on a Cobas system (Roche, Rotkreuz, Switzerland)
T50 (serum)	Nephelometry (Calciscon, Biel, Switzerland)[1]
Intact fibroblast growth factor 23 (serum)	iFGF23 ELISA kit (Kainos, Tokyo, Japan)
β-crosslaps (serum)	Electrochemiluminescence immunoassay (Roche, Rotkreuz, Switzerland)
Tartrate-resistant acid phosphatase isoform 5b (plasma)	Immunoassay (Immonudiagnostic Systems, Boldon, UK)
Calciprotein monomers (serum)	Fluorescent bisphosphonate probe-based assay[2,3]
Primary and secondary calciprotein particles (serum)	Fluorescent probe-based flow cytometric assay on an A50 Microflow Cytometer (Apogee, UK)[4,5]

References

1. Pasch A, Farese S, Gräber S, et al. Nanoparticle-based test measures overall propensity for calcification in serum. *J Am Soc Nephrol*. Oct 2012;23(10):1744-52. doi:10.1681/ASN.2012030240
2. Miura Y, Iwazu Y, Shiizaki K, et al. Identification and quantification of plasma calciprotein particles with distinct physical properties in patients with chronic kidney disease. *Sci Rep*. Jan 19 2018;8(1):1256. doi:10.1038/s41598-018-19677-4
3. Tiong MK, Holt SG, Ford ML, Smith ER. Serum Calciprotein Monomers and Chronic Kidney Disease Progression. *Am J Nephrol*. 2022;53(11-12):806-815. doi:10.1159/000526609
4. Smith ER, Hewitson TD, Cai MMX, et al. A novel fluorescent probe-based flow cytometric assay for mineral-containing nanoparticles in serum. *Sci Rep*. Jul 18 2017;7(1):5686. doi:10.1038/s41598-017-05474-y
5. Smith ER, Pan FFM, Hewitson TD, Toussaint ND, Holt SG. Effect of Sevelamer on Calciprotein Particles in Hemodialysis Patients: The Sevelamer Versus Calcium to Reduce Fetuin-A-Containing Calciprotein Particles in Dialysis (SCaRF) Randomized Controlled Trial. *Kidney Int Rep*. Sep 2020;5(9):1432-1447. doi:10.1016/j.ekir.2020.06.014

Supplementary Figure 1. Relationship between parathyroid hormone and T50 levels at an individual patient level

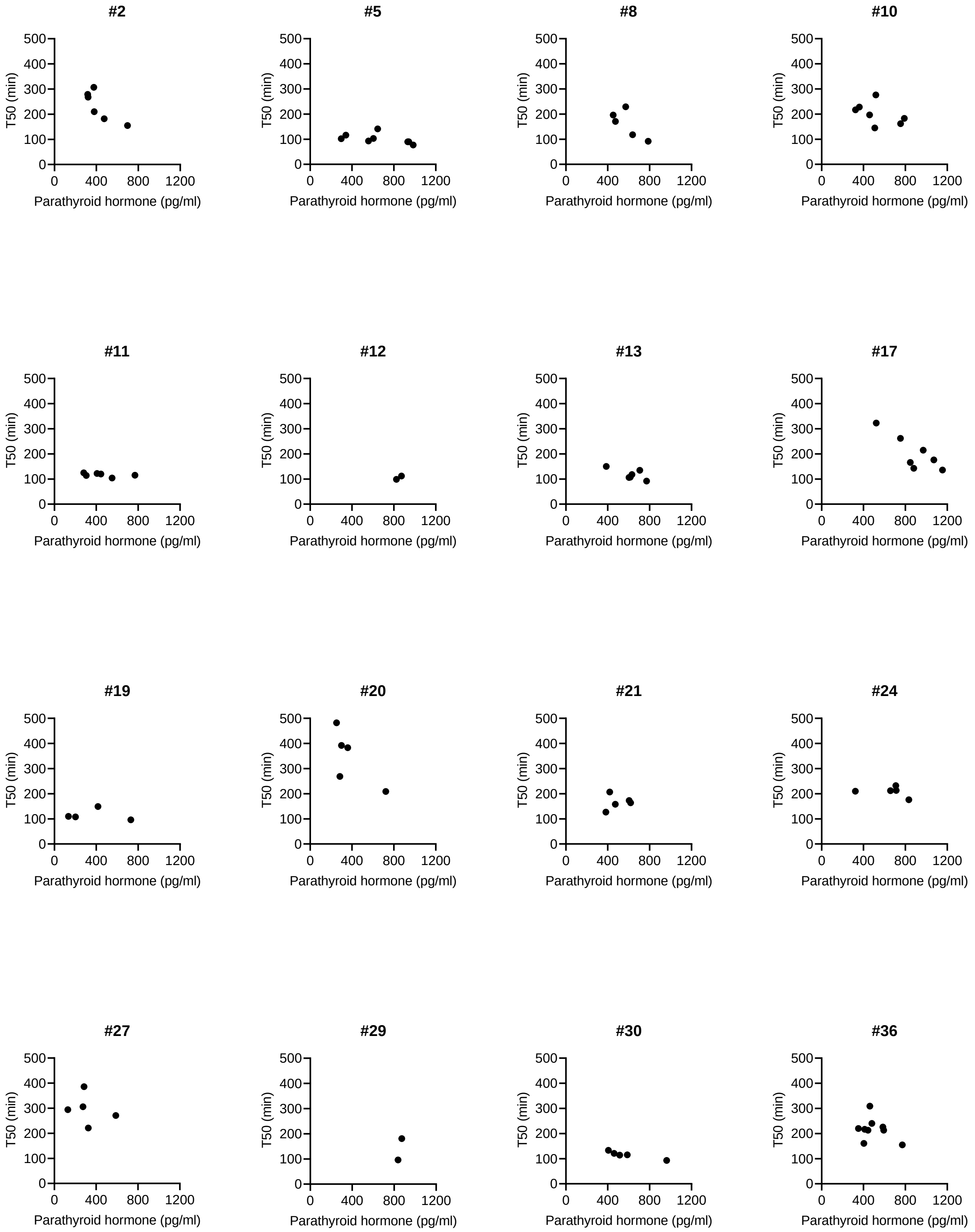

Supplementary Figure 1. Relationship between parathyroid hormone and T50 levels at an individual patient level

Chronic hemodialysis patients with secondary hyperparathyroidism were treated with escalating doses of etelcalcetide starting after a four- to twelve-week run-in/wash-out phase without the use of calcimimetics at a dose of 2.5 mg intravenously per dialysis session. Etelcalcetide dose was escalated every four weeks in 2.5 mg/dialysis session increments to a maximum dose of 15 mg thrice weekly until the end of study or until a pre-specified safety endpoint was reached. After completion of the 15 mg thrice-weekly phase or in case a safety endpoint was reached, etelcalcetide was discontinued and patients were followed for another eight weeks. All repeated measurements of parathyroid hormone and T50 during washout and treatment phases were used to explore their association at an individual patient level. Each panel represents one patient. # denotes patient ID.

Supplementary Figure 2. Correlation between dose-response slopes for T50 times and dose-response slopes for parathyroid hormone, serum phosphate, and calcium

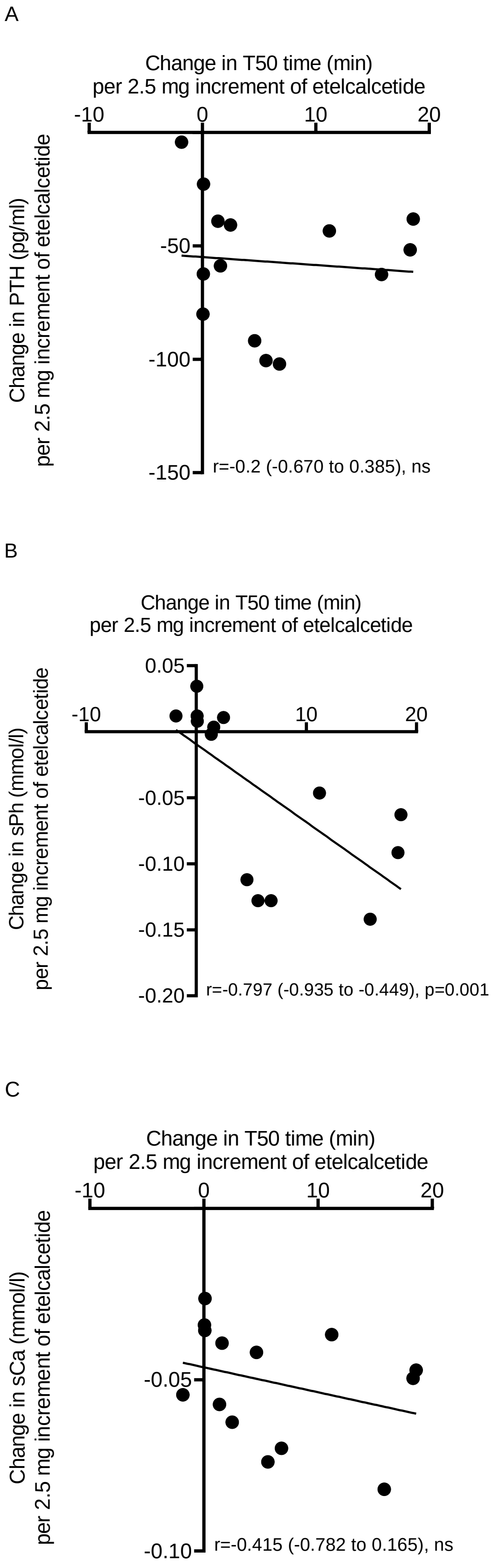

Supplementary Figure 2. Correlation between dose-response slopes for T50 times and dose-response slopes for parathyroid hormone, serum phosphate, and calcium

Chronic hemodialysis patients with secondary hyperparathyroidism were treated with escalating doses of etelcalcetide starting at a dose of 2.5 mg per dialysis session with increments of 2.5 mg per dialysis session every four weeks to a maximum dose of 15 mg thrice weekly or until a pre-specified safety endpoint was reached. After completion of the 15 mg thrice-weekly phase or in case a safety endpoint was reached, etelcalcetide was discontinued and patients entered an eight-week washout phase.

For Spearman correlation analyses, the slopes of the etelcalcetide dose-response curves including all measurements from baseline (after a four- to twelve-week washout phase) to the maximum tolerated etelcalcetide dose were derived by linear regression for each parameter.

PTH, parathyroid hormone; sPh, serum phosphate; sCa, serum calcium

Supplementary Figure 3. Correlation between dose-response slopes for serum phosphate and calcium and dose-response slopes for calciprotein monomers, primary and secondary calciprotein particles

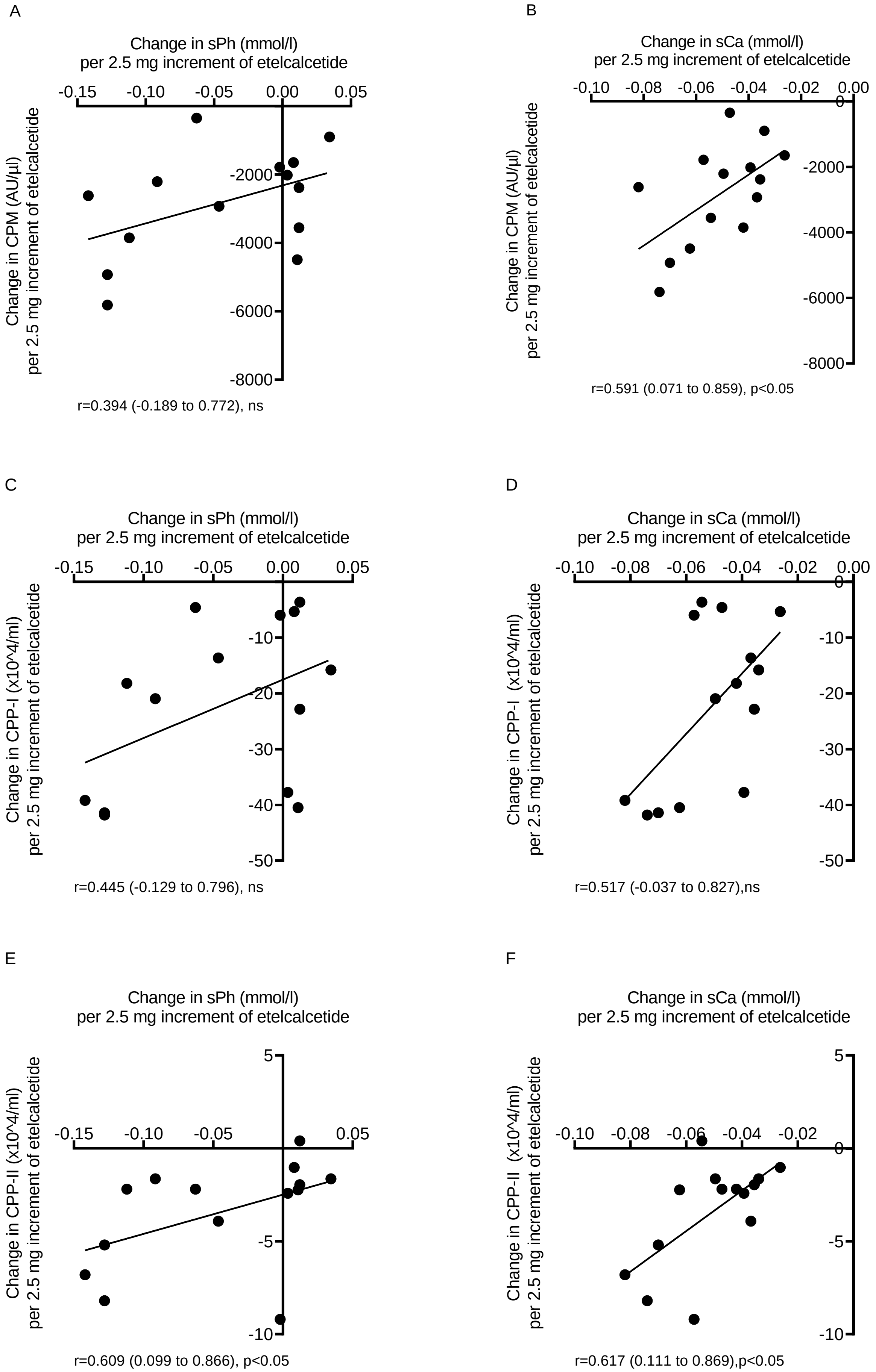
A
Change in sPh (mmol/l)
per 2.5 mg increment of etelcalcetide
-0.15
-0.10
-0.05
0.00
0.05
-2000
-4000
-6000
-8000
Change in CPM (AU/µl)
per 2.5 mg increment of etelcalcetide
r=0.394 (-0.189 to 0.772), ns
B
Change in sCa (mmol/l)
per 2.5 mg increment of etelcalcetide
-0.10
-0.08
-0.06
-0.04
-0.02
0.00
0
-2000
-4000
-6000
-8000
Change in CPM (AU/µl)
per 2.5 mg increment of etelcalcetide
r=0.591 (0.071 to 0.859), p<0.05
C
Change in sPh (mmol/l)
per 2.5 mg increment of etelcalcetide
-0.15
-0.10
-0.05
0.00
0.05
-10
-20
-30
-40
-50
Change in CPP-I (x10^4/ml)
per 2.5 mg increment of etelcalcetide
r=0.445 (-0.129 to 0.796), ns
D
Change in sCa (mmol/l)
per 2.5 mg increment of etelcalcetide
-0.10
-0.08
-0.06
-0.04
-0.02
0.00
0
-10
-20
-30
-40
-50
Change in CPP-I (x10^4/ml)
per 2.5 mg increment of etelcalcetide
r=0.517 (-0.037 to 0.827),ns
E
Change in sPh (mmol/l)
per 2.5 mg increment of etelcalcetide
5
-0.15
-0.10
-0.05
0.05
-5
-10
Change in CPP-II (x10^4/ml)
per 2.5 mg increment of etelcalcetide
r=0.609 (0.099 to 0.866), p<0.05
F
Change in sCa (mmol/l)
per 2.5 mg increment of etelcalcetide
5
-0.10
-0.08
-0.06
-0.04
-0.02
0
-5
-10
Change in CPP-II (x10^4/ml)
per 2.5 mg increment of etelcalcetide
r=0.617 (0.111 to 0.869),p<0.05

Supplementary Figure 3. Correlation between dose-response slopes for serum phosphate and calcium and dose-response slopes for calciprotein monomers, primary and secondary calciprotein particles

Chronic hemodialysis patients with secondary hyperparathyroidism were treated with escalating doses of etelcalcetide starting at a dose of 2.5 mg per dialysis session with increments of 2.5 mg per dialysis session every four weeks to a maximum dose of 15 mg thrice weekly or until a pre-specified safety endpoint was reached. After completion of the 15 mg thrice-weekly phase or in case a safety endpoint was reached, etelcalcetide was discontinued and patients entered an eight-week washout phase.

For Spearman correlation analyses, the slopes of the etelcalcetide dose-response curves including all measurements from baseline (after a four- to twelve-week washout phase) to the maximum tolerated etelcalcetide dose were derived by linear regression for each parameter. sPh, serum phosphate; sCa, serum calcium; CMP, calciprotein monomers; CPP-I, primary calciprotein particles; CPP-II, secondary calciprotein particles
